# Humanization of Tumor Stroma by Tissue Engineering as a Tool to Improve Squamous Cell Carcinoma Xenograft

**DOI:** 10.3390/ijms21061951

**Published:** 2020-03-12

**Authors:** Sara Guerrero-Aspizua, Andrea González-Masa, Claudio J. Conti, Marta García, Esteban Chacón-Solano, Fernando Larcher, Marcela del Río

**Affiliations:** 1 Department of Bioengineering, Universidad Carlos III de Madrid, 28911 Leganés, Spain; agonzalezmasa@gmail.com (A.G.-M.); cconti@ing.uc3m.es (C.J.C.); mgdiez@ing.uc3m.es (M.G.); echacon@ing.uc3m.es (E.C.-S.); fernando.larcher@ciemat.es (F.L.); mrnechae@ing.uc3m.es (M.d.R.); 2 Hospital Fundación Jiménez Díaz e Instituto de Investigación FJD, 28040 Madrid, Spain; 3 Epithelial Biomedicine Division. CIEMAT, 28040 Madrid, Spain; 4 Centre for Biomedical Network Research on Rare Diseases (CIBERER), U714, 28911 Madrid, Spain

**Keywords:** SCC, tissue engineering, stroma, CAF, tumor, xenografts

## Abstract

The role of stroma is fundamental in the development and behavior of epithelial tumors. In this regard, limited growth of squamous cell carcinomas (SCC) or cell-lines derived from them has been achieved in immunodeficient mice. Moreover, lack of faithful recapitulation of the original human neoplasia complexity is often observed in xenografted tumors. Here, we used tissue engineering techniques to recreate a humanized tumor stroma for SCCs grafted in host mice, by combining CAF (cancer associated fibroblasts)-like cells with a biocompatible scaffold. The stroma was either co-injected with epithelial cell lines derived from aggressive SCC or implanted 15 days before the injection of the tumoral cells, to allow its vascularization and maturation. None of the mice injected with the cell lines without stroma were able to develop a SCC. In contrast, tumors were able to grow when SCC cells were injected into previously established humanized stroma. Histologically, all of the regenerated tumors were moderately differentiated SCC with a well-developed stroma, resembling that found in the original human neoplasm. Persistence of human stromal cells was also confirmed by immunohistochemistry. In summary, we provide a proof of concept that humanized tumor stroma, generated by tissue engineering, can facilitate the development of epithelial tumors in immunodeficient mice.

## 1. Introduction

In the past decade, it has become clear that the behavior of a malignant tumor is related not only to the malignant cell itself, but also to the properties of the stromal microenvironment of the tumor [1,2]. In particular, in tumors of epithelial origin (i.e., carcinomas), the stroma appears to play an important role in the invasive properties of the tumor, its capacity to form metastasis as well as its evolution and prognosis [3].

The stroma of carcinomas is very complex concerning extracellular matrix (ECM) and cellular elements that contribute to tumor development. The most relevant cellular component of the tumor stroma are activated fibroblasts, characterized by an increased expression of α-smooth muscle actin (αSMA) and vimentin, a change from the fusiform to stellate shape, and enhanced ECM production [4]. They also alter the repertoire of paracrine signals including those related with immunoregulation [5]. These alterations appear to be the result of epigenetic changes that make these newly acquired properties irreversible. These altered fibroblasts, known as CAFs are somewhat similar to fibroblast present in cases of fibrosis, such as recessive dystrophic epidermolysis bullosa (RDEB) [6,7]. In addition to activated fibroblasts, the stroma of tumors displays a newly formed vascular network, which is recognized as essential in tumor development and metastatic behavior [8]. More recently, the focus of many laboratories has shifted to investigate the possible role of the ECM in cancer development and to the concomitant role of tumor fibroblasts to the synthesis and maintenance of this specific pro-tumorigenic ECM [6,7].

We have recently characterized a genetic signature of the CAF-like fibroblasts derived from three unrelated genodermatosis prone to develop cancer [9], stressing once more the importance of these cells in creating the proper environment, which contribute to the uncontrolled growth of transformed cells. However, despite the importance of in vitro experiments like ours and others, the actual growth of epithelial tumors and the contribution of the tumorigenic stroma are not always easy to recapitulate in xenografts. In this regard, the cancer experiments carried out in rodents, critical not only in the study of the mechanisms of carcinogenesis but also as an essential tool in the development of treatments, has a major deficiency. The original stroma of the tumor becomes replaced by stroma of the murine host leading to important changes in tumor behavior [10,11,12]. This is probably one of the major causes of disagreement between results in animal experiments and those achieved in clinical trials. In some cases, the stroma developed by the host is even unable to support the growth of epithelial tumors or cell lines derived from them, and therefore it is difficult to develop appropriate xenograft tumor models in some forms of cancer [6].

In this paper, we describe our efforts to humanize tumor stroma in an animal model, by tissue engineering techniques, with the goal of achieving tumors xenografts that mimics the actual behavior of human SCCs.

## 2. Results

**Figure 1:**
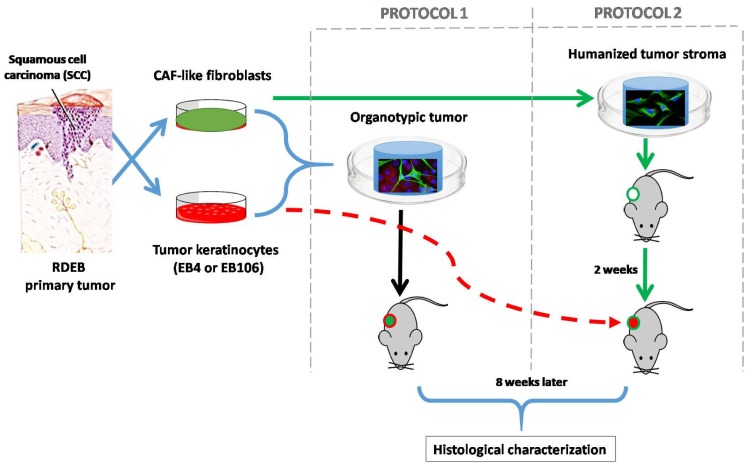
Tissue engineering humanized stroma for development of squamous cell carcinomas (SCC) in immunodeficient mice: two different approaches were followed. In protocol 1 simultaneous injection of tumoral cells and stroma (cancer associated fibroblasts (CAF)-like cells and biocompatible matrix) was performed, whereas in protocol 2 the generation of a humanized stroma was performed 2 weeks before the injection of the neoplastic keratinocytes. SCC-cells and stromal fibroblasts derived from recessive dystrophic epidermolysis bullosa (RDEB) patients were used during this experimentation, given their high predisposition of RDEB patients to develop very aggressive SCC. Moreover, the similarities of the molecular profile between RDEB dermal cells with that of CAFs, which promotes the invasion of tumor cells, has been widely studied [6,13,14,15,16,17]. In the first approach, to assess the tumorigenic potential of the SCC-derived cell lines used, immunodeficient mice were injected with tumor keratinocytes (EB4 or EB106), without the addition of exogenous stroma (2 × 10^6^ cells/animal. *n* = 3 in each experimental group). No tumor was detected after 60 days post-injection.

In order to provide a pro-tumorigenic humanized stroma to facilitate the growth of the SCC, we used two different approaches (Figure 1). In protocol 1 we use the simultaneous injection of tumoral and stromal cells embedded in a compatible biomaterial. Whereas in protocol 2, the generation of the humanized stroma was performed before the injection of the neoplastic cells. 

**Figure 2:**
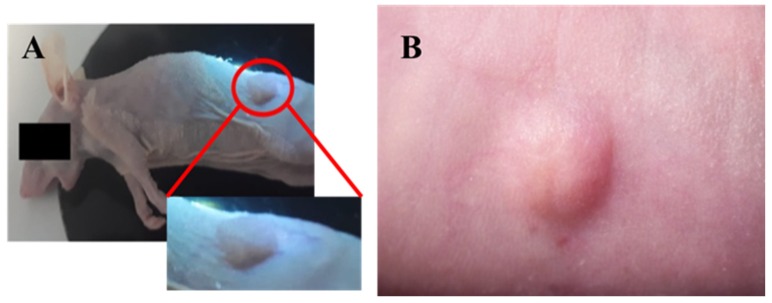
Macroscopic characterization of tumors developed in the presence of humanized stroma after 8 weeks of transplantation. (**a**) Tumor developed by protocol 1; (**b**) tumor developed by protocol2.

**Figure 3:**
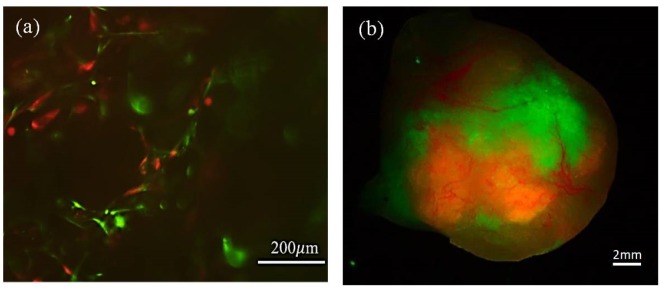
Persistence of tumor stromal cells in bioengineered SCC following protocols 1 and 2, respectively. (**a**) In vitro fluorescence microscope image from the cell culture, before transplantation, where both the EGFP-CAFs and RFP-SCC-keratinocytes are observed; scale bar: 200 μm. (**b**) In vivo3D fluorescence image obtained from the tumor, 8 weeks after implantation, using XLView Loupe. Scale bar: 2 mm.

In contrast, injection of cells according to protocols 1 and 2, led to tumor growth as evidenced by macroscopic visualization and fluorescence imaging (Figure 2 and Figure 3).

**Figure 4:**
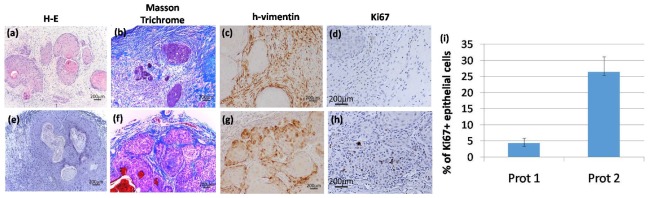
Histopathological analysis of representative tumors generated by different protocols of SCC stroma humanization. (**a–d**) Protocol 1; (**e–h**) protocol 2; (**a**) and (**e**)hematoxylin-eosin staining shows the structure of the tumors developed, as evidenced by the presence of tumor nests and cornified pearls and blood vessels in protocol 2; (**b**) and (**f**) Mason trichrome staining shows the different humanized tumoral stroma in both protocols;(**c**) and (**g**) show the human origin of fibroblasts surrounding the tumor structure by a h-vimentin staining; (**d**) and (**h**)show that tumors developed by protocol 2 have more proliferative cells as shown by a Ki67 staining.(**i**) Shows a quantification of the epithelial component of the tumor where ki67^+^ nuclei/100 epithelial cells is shown. For this calculation a total number of ten representative fields were included. Images are representative results for each group (*n* = 3). Scale bars 200 μm.

The efficiency of transfection of both cell types was evaluated during the in vitro cell culture of the graft in protocol 1, before implantation, by fluorescence images (Figure 3a) and the persistence of humanized stroma was observed after 8-weeks tumors was evaluated by the presence of green fluorescent cells macroscopically within the tumor mass in the flank of the mouse (Figure 3b). While the 3D macroscopic image showed that the stroma appeared to surround the tumor mass, the microscopic results showed that fibroblasts were intermingled with nests of tumor cells. Green (GFP) and red (RFP) fluorescence could be observed following both protocols. These results were confirmed by immunochemical staining of human specific vimentin in sections of the tumors as shown below (Figure 4c,g).

### Histological Characterization of the Bioengineered Tumor Stroma

A histopathological characterization was performed in all the tumors that were developed by protocol 1 in immunodeficient mice, these were characterized as well-differentiated SCC (Figure 4a) with a rich collagenous stroma (Figure 4b) and a noticeable residual presence of the artificial matrix. Despite the fact that the stroma in protocol 1 exhibited signs of survival and functionality, in some of the tumors, the epithelial cell tumor nests, were small and did not show significant proliferative activity (Figure 4d). 

In contrast, in the case of tumors (also well-differentiated SCC) developed using protocol 2 (Figure 4e–h) a clear increase in proliferative nests, as determined by Ki67 staining, was observed (Figure 4h), and the quantification of the epithelial part of the tumors showed an increase of Ki67+ cells, in the case of tumors developed by protocol 2 (Figure 4i). In fact, tumor architecture was much more similar to that observed in patient tumor biopsies (data not shown). The stroma, rich in thick and mature collagen fibers, intermixed with the tumor nests and surrounded the whole structure in a capsule-like arrangement (Figure 4f). To confirm that human CAF-like cells have not been replaced by murine fibroblast, we performed immunohistochemistry staining of tumor sections using a human-specific vimentin antibody (Figure 4c,g). Human vimentin positive fibroblasts, together with collagen-rich ECM, appear as the main components of the stroma surrounding the SCC cells nests. 

## 3. Discussion

The lack of SCC models with a humanized stroma represents an important drawback hampering and delaying the development of effective therapies of this common cancer [10,11,12,16]. In this regard, our study aimed to establish an experimental method that focused on the generation of a bioengineered stroma, capable of persisting and sustaining SCC growth. The importance of conditioning the environment for the successful growing of human carcinomas in immunodeficient mice is a very well-known phenomenon [18,19,20]. One of the early observations regarding this issue comes from studies with prostate carcinoma cells LNCaP, which under normal conditions, do not grow in vivo, unless the site of injection is preconditioned by implantation of matrigel or coinjection with human fibroblasts. Interestingly and pertinent to our work is the fact that not all fibroblasts have the capacity to facilitate the growth of LNCaP cells. In fact, normal dermal fibroblasts have no effect while fibroblasts obtained from prostate tumors were more efficient [21,22]. Models with other cell lines have also shown that stromal components may be required to grow or may accelerate the development of tumors such as breast cancer and non-small cell lung carcinomas [1,23,24,25].

In the case of skin SCC, a humanized stroma not only facilitates tumor growth, but also allows the manifestation of its full aggressiveness. In fact, one of the chief issues related with the skin SCC is the unusual behavior with regards to their metastatic potential. In the skin, more than 90% of SCC are relatively indolent and very rarely cause metastasis. However, around 5–10% of skin SCC tumors are extremely aggressive such as those from the oral mucosa, head and neck, esophagus, lung etc. Many of the aggressive SCC of the skin develops in areas with chronic ulcers as in hereditary conditions like epidermolysis bullosa [13,14,26]. The reason for this different behavior is still a subject of controversy and collaborative studies have recently shed light onto the matter [9,17,27,28,29]. Recently, attention has been focused on a possible role of the stroma. In this regard, the murine model appears to be not very useful because the stroma of transplanted tumors, including PDX models, is rapidly replaced by the host [10].

To cope with these methodological problems, two different strategies, adapted from previous approaches [10], have been used here to develop in vivo xenograft SCC organotypic models. In our attempt to generate a new model we took advantage of two SCC cell lines derived from patients with RDEB. Despite the aggressive nature of the primary tumors giving rise to the cell lines employed, the lack of a pre-conditioning of the stroma (i.e., cancer-prone humanized stroma) prevented tumor development. However, as previously found in human breast cancer and prostate tumor modeling [30], the pre-humanization and previous vascularization of the stroma seemed to be the most successful approach in SCC xenografting. In our case, the promotion of a vascularized bed prior SCC cells implantation showed a significant enhancement of tumor growth (Figure 4e–h). In contrast, when the SCC constructs were transplanted all together (cancer cells with stromal cells), tumor growth was not very efficient (Figure 4a–d).

In addition to the aggressive nature of the tumor cells itself, these evidences underscore the critical importance of the previous existence of a well-established human and vascularized niche able to recapitulate tumor complexity.

Bioengineering tools allow modifying the different components of a particular tumor helping to understand their influence on tumor aggressiveness. In this regard, and taking into account the immune component of the tumors and the activation of specific T-cell populations that has been described for other tumors such as ovarian cancer [31,32], the use of cancer and stromal cells from the same donors will be more appropriate than a heterologous system, as it has been described for aggressive tumors, such as neuroblastoma or pancreatic cancer [33,34,35]. In any case, the flexibility of our model allows the combination of different cell populations and genetic backgrounds and facilitates the study of the role of each component in the aggressiveness of the tumor.

Taken together our proof-of-concept study shows that tissue engineering tools applied to modeling the complexity of tumor environment, emerge as a powerful and promising technology, also in the context of personalized medicine, where the prognosis of each tumor can differ according to the underlying pathology, such as bullous diseases.

## 4. Materials and Methods

### 4.1. Cell Culture

CAF-like fibroblasts were obtained from biopsies of RDEB patients remnants of diagnosis donated for research under informed consent, after approval by the Ethics Committee of the University Hospital La Paz (Madrid, Spain) (approved date: 11 December 2018, approved number: HULP:PI-3426). 

The SCC-keratinocyte cell lines (EB4 and EB106) were kindly provided by Dr. Andrew P. South, Department of Dermatology & Cutaneous Biology (Thomas Jefferson University, Philadelphia, PA, USA) [36,37]. Both cell lines are RDEB-SCC derived, the EB4 line corresponds to the one with less tumorigenic potential while the EB106 cell line shows the most cancer-prone potential [38]. Cells were cultured in DMEM medium (GIBCO-BRL, Gaithersburg, MD, USA) 10% FBS, (Hyclone™ GE Healthcare Life Science, Chicago, IL, USA) and antibiotic-antimycotic, (E3473N; Thermo Fisher, Waltham, MA, USA), following standard protocols, and once embedded in a biocompatible matrix; the medium was replaced by DMEM medium with 10% FBS and 50 μg/mlascorbic acid (Sigma, St Louis, MO, USA).

### 4.2. Retroviral Gene Transfer

Human primary fibroblast (CAF-like cells) and keratinocyte cell line EB106 were transduced by incubation with a recombinant EGFP or RFP (tdTomato-expressing LZR-based, Clontech Laboratories, Inc. A Takara Bio Company, Mountain View, CA, USA) amphotropic retrovirus generated by transient transfection in 293T cells, as described in [39,40]. Transduction was performed at a titer of 1 × 10^6^ to 5 × 10^6^ CFU/mL together with Polybrene (8 mg/mL) for 6 h on two consecutive days. After the second infection, the cells were given fresh medium and allowed to reach 80–90% confluence. Cells were then trypsin-detached, resuspended in phosphate-buffered saline (PBS) 2% FBS, analyzed for EGFP or RFP expression, and sorted by fluorescence-activated cell sorting (FACS) on a FACStar PLUS flow cytometer (Becton Dickinson, San Jose, CA, USA).

### 4.3. Tissue Engineering for Modeling Cutaneous SCC

The protocol proposed by Patel et al. [10] was modified (Figure 1: Protocol 1 and Protocol 2) in order to optimize the period of time that is required for the development of the humanized ECM and minimize the number of cells used in the experiment.

Bioengineered scaffold was a combination of a biocompatible material (Spongostan Stand 50 × 70 × 10^2^ stuk, Johnson & Johnson Medical) and our fibrin gel, as previously described [41]. A mixture containing RDEB-CAFs (1.5 × 10^5^ /gel) was prepared with 350 μL of seeding medium (DMEM + 10% FBS), 1.5 μL of ascorbic acid (50 μg/mL), 7.5 μL of antifibrinolytic (Amchafibrin® 500mg; Fides Ecopharma S.A), 100 μL of fibrinogen from plasma cryoprecipitate of pig blood, and 50 μL of bovine thrombin (Sigma) diluted in CaCl_2_ (25 mM).The mixture was placed in one well of 24-cell culture plates (Costar®; Corning, New York, NY, USA), covering 1 cm^2^ of Spongostan™, and allowed to solidify in an incubator. After gelification (1 h), 1 mL of seeding medium with ascorbic acid was added and replaced every 48 h.

Cutaneous tumors were developed by using tissue engineering techniques, combining cells, scaffolds, and transplantation procedures following two different experimental protocols described as followed and schematized in Figure 1. 

#### 4.3.1. Protocol 1: Simultaneous Implantation of Tumor Cells and Stroma

It involves the simultaneous implantation of the stroma and the tumor cells (RDEB-CAFs and EB106 cancer keratinocytes) into the immunodeficient mice.

Three days post-ECM preparation, SCC-keratinocytes (1 × 10^6^ in 100 μL of seeding medium) were cultured in vitro inside the biocompatible scaffold through an injection. The external medium outside the biocompatible matrix-embedded was aspired prior to the incorporation of keratinocytes to prevent their suspension in the external media. The constructs were incubated for 1 h at 37 °C before 1 mL of seeding medium was added. Then, the organotypic tumors were cultured until transplantation into immunodeficient mice.

#### 4.3.2. Protocol 2: Previous Establishment of the Stroma and Posterior Introduction of Tumor Keratinocytes

The initial protocol was modified to develop a well-established and vascularized niche, previously to the implantation of cancer-prone keratinocytes (EB106) in order to enhance tumor growth and engraftment. Five days after ECM generation, the constructs were transplanted into immunodeficient mice and, once the pro-tumorigenic vascularized niche was developed (2 weeks post-transplantation of the construct), the SCC keratinocytes were incorporated.

In both protocols, 8 weeks post-transplantation, the mouse was euthanized, and the tumor tissue was dissected and submitted to the histology laboratory for embedding and sectioning.

### 4.4. Animals

For the grafting protocol immunodeficient Rj: NMRI-Foxn1nu (NMRI nu) female mice (6 weeks old) were used (Elevage Janvier, France). Mice were aseptically cleansed and grafted. They were housed for the duration of the experiment at the CIEMAT Laboratory Animal Facility (European Registration number ES280790000183; approved date: 17 April 2012) in pathogen-free conditions. All experimental procedures were carried out according to the European and Spanish laws. All procedures were approved by the Institutional Animal Care and Use Committee according to all legal regulations, and biosafety and bioethics guidelines. During the experiments, a minimum number of *n* = 3 animals were studied for each condition described in the results section.

### 4.5. Histological Characterization of Regenerated Bioengineered Tumors

Formalin-fixed paraffin sections (4–6 μm) were used for standard histological and IHC analyses. To determine tissue architecture, sections were stained with hematoxylin and eosin (Gill 2 Hematoxylin and Eosin Y alcoholic; Thermo Shandon, Altrincham, UK) following a standard Masson trichrome (Sigma-Aldrich kit) staining protocol. Immunohistochemistry was carried out using an anti-Ki-67 rabbit monoclonal antibody (Neomarkers clone SP6) for cell proliferation and human specific anti-vimentin antibody (Biogenex #AM074-10M, USA) following the manufacturer’s protocol. Specific biotinylated secondary antibodies (Jackson ImmunoResearch Laboratories; West Grove, PA, USA) were used and immunoperoxidase staining (Vectastain ABC kit; Vector Laboratories Inc., Burlingame, CA, USA) was performed using standard procedures. Sections were counterstained with hematoxylin and dehydrated in water solution containing increasing percentages of ethanol. Finally, the slides were placed 15 min in histoclear (National diagnostic, Atlanta, GA, USA) and mounted. Images were taken using an Olympus Bx41 microscope and digital camera.

## Abbreviations

CAF	Cancer associated fibroblasts
ECM	Extracellular matrix
EGFP	Enhanced green fluorescent protein
RDEB	Recessive dystrophic epidermolysis bullosa
RFP	Red fluorescent protein
SCC	Squamous cell carcinoma
SMA	Smooth muscle actin

## References

[1] Bremnes R.M., Donnem T., Al-Saad S., Al-Shibli K., Andersen S., Sirera R., Camps C., Marinez I., Busund L.T. (2011). The role of tumor stroma in cancer progression and prognosis: Emphasis on carcinoma-associated fibroblasts and non-small cell lung cancer. J. Thorac. Oncol..

[2] De Wever O., Mareel M. (2003). Role of tissue stroma in cancer cell invasion. J. Pathol..

[3] Chan T.S., Shaked Y., Tsai K.K. (2019). Targeting the Interplay Between Cancer Fibroblasts, Mesenchymal Stem Cells, and Cancer Stem Cells in Desmoplastic Cancers. Front. Oncol..

[4] Lindner T., Loktev A., Giesel F., Kratochwil C., Altmann A., Haberkorn U. (2019). Targeting of activated fibroblasts for imaging and therapy. EJNMMI Radiopharm. Chem..

[5] Ao M., Franco O.E., Park D., Raman D., Williams K., Hayward S.W. (2007). Cross-talk between paracrine-acting cytokine and chemokine pathways promotes malignancy in benign human prostatic epithelium. Cancer Res..

[6] Ng Y.Z., Pourreyron C., Salas-Alanis J.C., Dayal J.H., Cepeda-Valdes R., Yan W., Wright S., Chen M., Fine J.D., Hogg F.J. (2012). Fibroblast-derived dermal matrix drives development of aggressive cutaneous squamous cell carcinoma in patients with recessive dystrophic epidermolysis bullosa. Cancer Res..

[7] Sahai E., Astsaturov I., Cukierman E., DeNardo D.G., Egeblad M., Evans R.M., Fearon D., Greten F.R., Hingorani S.R., Hunter T. (2020). A framework for advancing our understanding of cancer-associated fibroblasts. Nat. Rev. Cancer.

[8] Casanova M.L., Larcher F., Casanova B., Murillas R., Fernandez-Acenero M.J., Villanueva C., Martinez-Palacio J., Ullrich A., Conti C.J., Jorcano J.L. (2002). A critical role for ras-mediated, epidermal growth factor receptor-dependent angiogenesis in mouse skin carcinogenesis. Cancer Res..

[9] Chacon-Solano E., Leon C., Diaz F., Garcia-Garcia F., Garcia M., Escamez M.J., Guerrero-Aspizua S., Conti C.J., Mencia A., Martinez-Santamaria L. (2019). Fibroblast activation and abnormal extracellular matrix remodelling as common hallmarks in three cancer-prone genodermatoses. Br. J. Dermatol..

[10] Patel G.K., Yee C.L., Yuspa S.H., Vogel J.C. (2012). A humanized stromal bed is required for engraftment of isolated human primary squamous cell carcinoma cells in immunocompromised mice. J. Invest. Dermatol..

[11] Hidalgo M., Amant F., Biankin A.V., Budinska E., Byrne A.T., Caldas C., Clarke R.B., de Jong S., Jonkers J., Maelandsmo G.M. (2014). Patient-derived xenograft models: An emerging platform for translational cancer research. Cancer Discov..

[12] Malaney P., Nicosia S.V., Dave V. (2014). One mouse, one patient paradigm: New avatars of personalized cancer therapy. Cancer Lett..

[13] Martins V.L., Vyas J.J., Chen M., Purdie K., Mein C.A., South A.P., Storey A., McGrath J.A., O’Toole E.A. (2009). Increased invasive behaviour in cutaneous squamous cell carcinoma with loss of basement-membrane type VII collagen. J. Cell Sci..

[14] Kim M., Murrell D.F. (2015). Update on the pathogenesis of squamous cell carcinoma development in recessive dystrophic epidermolysis bullosa. Eur. J. Dermatol..

[15] Martins V.L., Caley M.P., Moore K., Szentpetery Z., Marsh S.T., Murrell D.F., Kim M.H., Avari M., McGrath J.A., Cerio R. (2016). Suppression of TGFbeta and Angiogenesis by Type VII Collagen in Cutaneous SCC. J. Natl Cancer Inst..

[16] Mittapalli V.R., Madl J., Loffek S., Kiritsi D., Kern J.S., Romer W., Nystrom A., Bruckner-Tuderman L. (2016). Injury-Driven Stiffening of the Dermis Expedites Skin Carcinoma Progression. Cancer Res..

[17] Cho R.J., Alexandrov L.B., den Breems N.Y., Atanasova V.S., Farshchian M., Purdom E., Nguyen T.N., Coarfa C., Rajapakshe K., Prisco M. (2018). APOBEC mutation drives early-onset squamous cell carcinomas in recessive dystrophic epidermolysis bullosa. Sci. Transl. Med..

[18] Coltrini D., Ronca R., Belleri M., Zardi L., Indraccolo S., Scarlato V., Giavazzi R., Presta M. (2009). Impact of VEGF-dependent tumour micro-environment on EDB fibronectin expression by subcutaneous human tumour xenografts in nude mice. J. Pathol..

[19] Mollo M.R., Antonini D., Cirillo L., Missero C. (2016). Research Techniques Made Simple: Skin Carcinogenesis Models: Xenotransplantation Techniques. J. Invest. Dermatol..

[20] Yoshida G.J. (2020). Applications of patient-derived tumor xenograft models and tumor organoids. J. Hematol. Oncol..

[21] Chung L.W. (1991). Fibroblasts are critical determinants in prostatic cancer growth and dissemination. Cancer Metastasis Rev..

[22] Stephenson R.A., Dinney C.P., Gohji K., Ordonez N.G., Killion J.J., Fidler I.J. (1992). Metastatic model for human prostate cancer using orthotopic implantation in nude mice. J. Natl. Cancer Inst..

[23] Bhowmick N.A., Neilson E.G., Moses H.L. (2004). Stromal fibroblasts in cancer initiation and progression. Nature.

[24] Orimo A., Gupta P.B., Sgroi D.C., Arenzana-Seisdedos F., Delaunay T., Naeem R., Carey V.J., Richardson A.L., Weinberg R.A. (2005). Stromal fibroblasts present in invasive human breast carcinomas promote tumor growth and angiogenesis through elevated SDF-1/CXCL12 secretion. Cell.

[25] Ma Y., Lin Z., Fallon J.K., Zhao Q., Liu D., Wang Y., Liu F. (2015). New mouse xenograft model modulated by tumor-associated fibroblasts for human multi-drug resistance in cancer. Oncol. Rep..

[26] Guerra L., Odorisio T., Zambruno G., Castiglia D. (2017). Stromal microenvironment in type VII collagen-deficient skin: The ground for squamous cell carcinoma development. Matrix Biol..

[27] Guerrero-Aspizua S., Conti C.J., Escamez M.J., Castiglia D., Zambruno G., Youssefian L., Vahidnezhad H., Requena L., Itin P., Tadini G. (2019). Assessment of the risk and characterization of non-melanoma skin cancer in Kindler syndrome: Study of a series of 91 patients. Orphanet J. Rare Dis..

[28] Condorelli A.G., Dellambra E., Logli E., Zambruno G., Castiglia D. (2019). Epidermolysis Bullosa-Associated Squamous Cell Carcinoma: From Pathogenesis to Therapeutic Perspectives. Int. J. Mol. Sci..

[29] Atanasova V.S., Russell R.J., Webster T.G., Cao Q., Agarwal P., Lim Y.Z., Krishnan S., Fuentes I., Guttmann-Gruber C., McGrath J.A. (2019). Thrombospondin-1 Is a Major Activator of TGF-beta Signaling in Recessive Dystrophic Epidermolysis Bullosa Fibroblasts. J. Invest. Dermatol..

[30] Kuperwasser C., Chavarria T., Wu M., Magrane G., Gray J.W., Carey L., Richardson A., Weinberg R.A. (2004). Reconstruction of functionally normal and malignant human breast tissues in mice. Proc. Natl. Acad. Sci. USA.

[31] Want M.Y., Konstorum A., Huang R.Y., Jain V., Matsueda S., Tsuji T., Lugade A., Odunsi K., Koya R., Battaglia S. (2019). Neoantigens retention in patient derived xenograft models mediates autologous T cells activation in ovarian cancer. Oncoimmunology.

[32] Gitto S.B., Kim H., Rafail S., Omran D.K., Medvedev S., Kinose Y., Rodriguez-Garcia A., Flowers A.J., Xu H., Schwartz L.E. (2020). An autologous humanized patient-derived-xenograft platform to evaluate immunotherapy in ovarian cancer. Gynecol. Oncol..

[33] Williams J.A. (2018). Using PDX for Preclinical Cancer Drug Discovery: The Evolving Field. J. Clin. Med..

[34] Braekeveldt N., von Stedingk K., Fransson S., Martinez-Monleon A., Lindgren D., Axelson H., Levander F., Willforss J., Hansson K., Ora I. (2018). Patient-Derived Xenograft Models Reveal Intratumor Heterogeneity and Temporal Stability in Neuroblastoma. Cancer Res..

[35] Wang Y., Cui J., Wang L. (2019). Patient-derived xenografts: A valuable platform for clinical and preclinical research in pancreatic cancer. Chin. Clin. Oncol..

[36] Purdie K.J., Pourreyron C., South A.P. (2011). Isolation and culture of squamous cell carcinoma lines. Methods Mol. Biol..

[37] Atanasova V.S., Pourreyron C., Farshchian M., Lawler M., Brown C.A.t., Watt S.A., Wright S., Warkala M., Guttmann-Gruber C., Hofbauer J.P. (2019). Identification of Rigosertib for the Treatment of Recessive Dystrophic Epidermolysis Bullosa-Associated Squamous Cell Carcinoma. Clin. Cancer Res..

[38] Inman G.J., Wang J., Nagano A., Alexandrov L.B., Purdie K.J., Taylor R.G., Sherwood V., Thomson J., Hogan S., Spender L.C. (2018). The genomic landscape of cutaneous SCC reveals drivers and a novel azathioprine associated mutational signature. Nat. Commun..

[39] Larcher F., Del Rio M., Serrano F., Segovia J.C., Ramirez A., Meana A., Page A., Abad J.L., Gonzalez M.A., Bueren J. (2001). A cutaneous gene therapy approach to human leptin deficiencies: Correction of the murine ob/ob phenotype using leptin-targeted keratinocyte grafts. FASEB J..

[40] Del Rio M., Larcher F., Serrano F., Meana A., Munoz M., Garcia M., Munoz E., Martin C., Bernad A., Jorcano J.L. (2002). A preclinical model for the analysis of genetically modified human skin in vivo. Hum. Gene. Ther..

[41] Meana A., Iglesias J., Del Rio M., Larcher F., Madrigal B., Fresno M.F., Martin C., San Roman F., Tevar F. (1998). Large surface of cultured human epithelium obtained on a dermal matrix based on live fibroblast-containing fibrin gels. Burns.

